# Supplementary material for: Are carbon emissions trading and green financial instruments synergistic? -Comprehensive quantitative research based on content analysis
Source: PLoS One. 2024 Mar 7;19(3):e0298601. doi: 10.1371/journal.pone.0298601 (PMC10919601; doi:10.1371/journal.pone.0298601)
Supplement: S1 Data — (ZIP) [file pone.0298601.s001.zip › Data/地级市Z_F工作报告.zip!/╒π╜¡/║■╓▌╩╨/└┤╫╘╣1⁄2╓┌║┼í╛┬φ┐╦╩2╛▌═°í┐_2018.docx]

各位代表：

现在，我代表湖州市人民政府，向大会报告工作，请予审议，并请市政协委员和其他列席人员提出意见。

一、 2017年主要工作

回顾本届市政府开局之年，在省委、省政府和市委的坚强领导下，在市人大、市政协等各方面的监督和支持下，我们紧紧依靠全市人民，以迎接党的十九大胜利召开、学习贯彻党的十九大精神为动力，按照市第八次党代会作出的决策部署，自觉践行新发展理念，持续深化供给侧结构性改革，全力投入“四新”主题实践，认真落实“六重”工作，紧盯26件重大事项，统筹推进经济社会发展，较好地完成了市八届人大一次会议确定的主要目标任务。地区生产总值达到2476.1亿元，增长8.5%，增幅居全省第二位；财政总收入达到408.9亿元，其中地方财政收入237.4亿元，分别增长13.3%和15.2%，增幅居全省第二和第一位；城镇、农村居民人均可支配收入分别达到49934元和28999元，增长9%和9.4%，增幅居全省第二和第四位；十方面民生实事项目全面完成。

过去一年，面对重重考验，我们昂扬奋进，抓了一批关系全局、关乎长远、关及根本的大事、要事、实事、难事，加快赶超、实现“两高”迈出了全新步伐。一年来，我们从湖州发展最紧缺的事情抓起，深度对接国家战略，陆续获得了“中国制造2025”试点示范城市、国家绿色金融改革创新试验区、国家生态文明建设示范市等多个国家级试点示范；一年来，我们从湖州发展最紧迫的事情抓起，大力优化基础环境，沪苏湖高铁项目取得突破性进展，2017湖商发展大会隆重举办，一批新的万亩大平台打造成形，招商引资、招才引智、扶持实体经济等政策和工作力度不断加大；一年来，我们从湖州发展最紧要的事情抓起，全力投入攻坚克难，全面打响了“最多跑一次”改革、城中村改造和建设项目遗留问题清零、全域剿灭劣Ⅴ类水等攻坚战。尤其倍感振奋的是，全市上下众志成城、决战决胜，以优异成绩成功创建了全国文明城市。

（一）坚持提质增效，实体经济稳中向好

产业转型升级迈出新步伐。全面推进“中国制造2025”试点示范城市建设，设立15亿元的专项资金，以绿色制造、智能制造引领工业转型升级，规上工业增加值增长8.7%，其中战略性新兴产业、高新技术产业、装备制造业分别增长9.5%、 10.4%和14.5%，新能源汽车、美妆时尚、地理信息等新增长点不断发展壮大。开展“四无”企业（作坊）整治，淘汰和整治“低小散”企业及作坊3058家。服务业继续保持增速较快、占比提升的发展势头，增加值增幅达到10%。旅游总收入突破千亿元，安吉灵峰旅游度假区跻身国家级行列；网络零售额达到423.6亿元；内河集装箱吞吐量突破35万标箱，居全国同类型港口首位。我市在农业现代化发展水平综合评价中连续第四年居全省首位，现代生态循环农业试点市建设通过省级验收。新建成粮食生产功能区4.7万亩，新增省级现代农业园区4个、农业品牌25个，累计建成产值10亿元以上的省级示范性农业全产业链10条。

项目引进推进实现新突破。完善招商引资工作机制，精准对接浙商、外资、央企等资本，浙商回归省外到位资金531.1亿元，实到外资10.5亿美元，引进首期固定资产投资超10亿元的“大好高”项目22个，游侠汽车超级工厂、唯品会华东中心、万邦德“绿色智造”新材料、天士力大健康产业基地等项目签约落户。全力扩大有效投资，完成固定资产投资1731亿元，其中市重点建设项目完成投资超过1000亿元，列入省重点建设项目、“411”重大项目、重大产业项目的数量均居全省首位，太湖龙之梦、长龙山抽水蓄能电站等项目加快推进。启动长兴绿色智能制造产业园、吴兴高新区、南浔经济开发区、太湖度假区长东片区等万亩大平台规划建设，4个特色小镇新列入省级创建和培育名单。

企业培大育强取得新进展。深入实施“金象金牛”大企业、“高技术高成长”企业和科技型小微企业成长等培育计划，“双金”企业达到16家、“双高”企业达到126家， 9家企业入围中国民企制造业500强，新增上市公司7家，累计达到30家，完成“个转企” 1506家、“小升规” 358家、“下升上” 284家。出台新一轮降本减负政策，为企业减轻负担67.5亿元。深化质量标准品牌建设，新增国家标准40项、“浙江制造”品牌培育企业37家。推动企业全方位对接国际资源和市场，完成进出口总额772.1亿元，其中出口681.4亿元，分别增长14.6%和14.7%，服务贸易规模突破百亿元，新增省级国际产业合作园2家。

（二）坚持改革创新，发展活力不断迸发

“最多跑一次”等改革全面推进。群众和企业到政府办事“跑一次”和“零上门”事项分别占到全部办事事项的97.8%和59%，市、县区两级办事事项分别减少47.5%和38.9%。“多证合一”改革顺利推进，办理“多证合一”营业执照4.9万份。开展企业投资项目承诺报备制和发改委“一窗服务”改革试点；实施“区域能评＋区块能耗标准”“区域环评＋环境标准”制度；全省首块“标准地”在德清成交，供地模式在全市推广。调整市对区财政体制，财力进一步向基层倾斜。深化国有企业改革与发展，市属四大国企集团净资产增长20.1%，县区国企重组步伐加快。完成盐业体制改革。启动“五未”土地处置专项行动，促进土地节约集约利用，新增建设用地指标2.65万亩，消化批而未供土地2.15万亩，盘活存量建设用地1.35万亩。农村土地承包经营权确权登记颁证基本完成，流转土地经营权证、宅基地及房屋登记发证实现应发尽发，集体经营性建设用地入市改革不断深化。

绿色金融改革创新扎实开展。全方位推进国家绿色金融改革创新试验区建设，设立10亿元的专项资金，建立绿色金融专营机构12家，开发绿色金融产品94只，太湖绿色金融小镇挂牌成立。引导资金支持先进制造业等实体经济发展，金融机构贷款余额3281亿元，增长19%，增幅居全省第一位；完成直接融资802.3亿元。政府产业引导基金发挥实效，子基金规模达到232.8亿元，项目投资总额73.8亿元。切实防控金融风险，银行不良贷款率进一步降低至0.95%，信贷资产质量居全省第二位，企业资金链担保链等领域风险得到有效管控。加强政府性债务管理，压缩负债规模，壮大可用财力，市级及各县区政府债务率全面下降。

创新创业氛围日益浓厚。深入实施“人才新政”，打出聚引人才、促进创新、推动创业的组合拳，新引进各类人才2.9万名、“南太湖精英计划”领军型创新创业团队和人才项目211个、省领军型创新创业团队5个，入选“国千”人才13名、“省千”人才19名，扶持大学生创业3727人，新增高技能人才1.8万名。大力支持众创空间建设，新认定国家级和省级众创空间26家。推进国家科技创新服务体系建设试点，深化政产学研合作，启动地校合作四年行动计划，新增国家重点扶持高新技术企业96家、发明专利授权量2190件，3家产业创新服务综合体列入省级创建和培育名单。全面启动湖州科技城建设，加快莫干山国家级高新区等创新平台发展，长兴新能源装备、安吉绿色制造高新技术产业园区成为创建型省级高新区。国家创新型试点城市建设通过验收评估。我市在全省党政领导科技进步目标责任制考核中连续第五年获评为优秀单位。（三）坚持“两山”引领，城乡面貌显著改善

生态文明建设继续走在前列。入选全国首批“绿水青山就是金山银山”实践创新基地，成为全国水生态文明城市。制定党政领导干部生态环境损害责任追究办法，开展乡镇领导干部自然资源资产离任审计试点，划定生态保护红线，生态文明标准体系全面建立。扎实整改中央环保督察发现的问题。 9905名河长带头治水，在全省率先剿灭劣Ⅴ类水，完成河道清淤1200万方，商品温室龟鳖养殖实现全域清零， 77个县控及以上断面水质全部达到或优于Ⅲ类。持续开展大气污染防治，实施电能替代项目230个，火热电企业全部达到国家清洁化排放标准， 10蒸吨以下高污染燃料小锅炉全部淘汰，市区空气质量优良率68.5%，提高2.9个百分点，PM2.5平均浓度每立方米42微克，下降10.6%。矿山开采量稳步下降，完成废弃矿山治理1.5万亩。“两路两侧”“四边三化”环境治理继续深化。扎实开展综合治理毁林（竹）专项行动，完成造林更新7981亩、平原绿化1.8万亩，建设珍贵彩色健康森林7.9万亩。

文明城市创建工作取得硕果。在创建全国文明城市过程中，全民总动员、全员齐上阵，“路长制”“红马甲”遍布湖城、成为常态，累计有70多万人次的志愿者走上街头、服务社会，城市精神全面彰显，文明张力充分激发。扎实开展“十整治一完善”百日攻坚行动，累计整治老旧小区270个、农贸市场81家，新增停车位1.8万余个，学府路“垃圾街”、银瑞小商品市场等一批老大难问题得到彻底解决，市容市貌明显改善，城市品质切实提升。全面开展中心城市生活垃圾分类收集处置，推进“厕所革命”，入选国家公交都市创建城市。禁止销售燃放烟花爆竹初战告捷。长兴荣膺县级全国文明城市。全市首批43个小城镇完成环境综合整治，城镇危旧住宅房屋治理改造三年任务提前完成。安吉入列全省首批“无违建县”。

城乡建设水平加快提升。全面完成建设项目遗留问题清零，在此基础上城中村改造攻坚拆除2.6万户、1042万平方米，均通过房票等形式妥善安置，为民生增添了福祉，为发展腾出了空间。全市核发工程规划许可项目827个，增长53.1%。南太湖滨湖一体化高质量推进，太湖度假区、长兴图影、吴兴东部新区、湖州开发区、南浔中心城区等板块融合发展进程加快。湖山大道、湖长二通道等项目有序推进，南太湖湿地奥体公园投入使用。持续推进交通大建设大发展，商合杭高铁、杭宁高速公路改扩建、申嘉湖高速公路西延、京杭运河整治等项目加快实施，杭州绕城高速公路西复线、长湖申航道西延等项目开工建设。四大重点水利工程基本建成，城乡防洪能力大幅提升。美丽乡村建设提标升级，市级美丽乡村覆盖率达到89.6%，创建56个精品村和11个示范乡镇，农村生活垃圾分类处理基本实现全覆盖，创建美丽乡村路355.8公里。与浙江大学合作共建美丽乡村不断深化。（四）坚持民生为本，社会发展持续进步

社会保障水平稳步提高。以大学生、城镇零就业家庭和低收入农户等为重点，精准提供就业服务，城镇新增就业10.6万人，登记失业率2.22%。深入实施全民参保计划，基本养老保险待遇水平进一步提高。全面落实全市统一的城乡居民基本医疗保险和大病保险政策，实施医保个人账户家庭共济。加快养老服务体系建设，每千名老人拥有养老床位增加到36.4张。市老年大学新建项目开工建设。健全社会救助体系，全面实施支出型贫困家庭基本生活救助，低保标准进一步提高。扩大住房公积金制度覆盖面,新增缴存职工8.4万人。关爱道德模范工作切实加强，妇女、儿童事业加快发展，残疾人共享全面小康工程深入推进。

社会事业发展更加协调。新高考平稳有序实施，义务教育标准化学校覆盖率达到95%，学前教育“安吉游戏”模式享誉国际，职业教育、特殊教育加快发展，湖州师院获得硕士学位授予权，浙工大德清校区开工建设。南浔区创建成为省教育基本现代化区。全面启动中心城区医疗资源布局调整，浙北医学中心等项目加快建设。深化医药卫生体制改革，药品供应保障机制改革取得突破，公立医院薪酬制度改革稳步推进，基层医疗卫生服务体系更加完善，“双下沉、两提升”工程取得更多实效。长兴县成为国家级综合医改示范县。全国基层中医药工作先进县区实现全覆盖。完成红十字应急救护普及培训10.5万人。公共文化服务体系不断完善，综合性公共数字文化服务平台“湖上云”上线运行，新建成农村文化礼堂60家，桑基鱼塘系统被认定为全球重要农业文化遗产。第十六届省运会筹备工作全面推进，群众体育蓬勃发展，省级体育强县区实现全覆盖。档案、地方志等事业加快发展。

社会治理体系不断完善。“七五”普法深入实施，公共法律服务体系建设持续加强，社会信用体系建设加快推进，有力推动了法治湖州建设进程。加快建立全方位、立体化、信息化社会治安防控体系，依法严厉打击工程建设、环境保护、食品药品安全等领域违法犯罪活动，成功破获、练市等地重特大命积案，刑事案件发生数下降31.4%。严格落实安全生产主体责任、监管责任，全方位排查整治重点行业领域事故隐患，安全生产事故起数下降25.8%。全面建成“基层治理四平台”、县区矛盾纠纷多元化解中心，吴兴区入选全国首批街道服务管理创新实验区。重大安保任务圆满完成，平安湖州建设有望实现“十一连冠”。深化国防动员和全民国防教育，启动新一轮双拥模范城创建工作。民族、宗教、外事侨务、对台等工作进一步加强。

（五）坚持担当实干，政府自身建设切实加强

行政效能建设扎实推进。大力倡导分内的事认真干、交叉的事主动干、分外的事愉快干，坚持一般事项答复、请示件办理、群众来信批阅“三个不过夜”，政府工作效率持续提升。坚持精细化抓推进，年初确定全年重点工作清单，市政府常务会议定期听取26件重大事项进展情况汇报，并进行打分评价考核。深化“互看互比”活动，对在全省排名靠后的工作，及时研究和落实改进举措，努力争先进位。完善统一政务咨询投诉举报平台，受理群众诉求84.2万件，基本得到有效办结。

法治政府建设不断深化。加强政府立法工作，出台市政府规章制定程序规定，提请市人大常委会审议2部地方性法规草案。全面清理行政规范性文件。严格落实行政执法责任制，加强执法主体资格管理。启动行政复议体制改革，推动行政机关主要负责人出庭应诉，成立全省首个设区市行政争议调解中心。全面加强政府信息公开，建立市人大代表、市政协委员列席市政府常务会议制度，形成政府决策“开门听建议”常态化机制。市政府咨询委、法律顾问组等智库建设进一步加强。认真参与人大代表问政会、政协委员与市长“面对面”、“电视问政”等活动，办结市人大代表建议191件、政协提案293件。

党风廉政建设持续加强。认真履行市政府党组主体责任，全面从严加强政府系统党的建设，扎实推进“两学一做”学习教育常态化制度化，严格落实中央八项规定精神，严守各项纪律规矩。支持纪检监察机关监督执纪问责，重点环节、重点领域廉政风险防控工作进一步加强。强化机构编制管理，规范编外用人。整合建立公共资源交易集中监管体制，初步形成全市统一的公共资源交易平台体系。切实管好用好财政资金，开展审计体制改革试点，审计监督覆盖面进一步扩大。坚持惩治腐败无禁区、全覆盖、零容忍，反腐败斗争压倒性态势已经形成并巩固发展。

各位代表！本届政府工作开局良好。看似寻常最奇崛，成如容易却艰辛。这些成绩的取得，是省委、省政府和市委正确领导的结果，是全市上下、各个方面团结拼搏的结果。在此，我代表市人民政府，向包括全体人大代表、政协委员、离退休老同志在内的全市人民，向各民主党派、工商联、人民团体和社会各界人士，向驻湖人民解放军和武警部队官兵，向所有关心和支持湖州发展的海内外朋友，表示衷心的感谢，致以崇高的敬意！

在看到成绩的同时，我们也清醒地认识到，发展中不平衡不充分的现象依然突出，工作中问题和短板依然不少。主要是：产业结构调整还不够快，高端创新要素、重大产业项目不够多，亩均产出水平不高，节能降耗压力不小，高质量发展任重道远；教育、医疗、养老等社会公共服务水平与群众需求相比还有较大差距，文明城市的长效管理需要加强，公共安全方面仍有不少薄弱环节；生态环境领域短板不少，空气质量、水体感观仍有不小差距；互联网金融、地方政府性债务等领域存在一定的风险隐患，防范和化解重大风险进入攻坚期；政府转职能、提效能的任务仍然艰巨，少数干部担当精神不强，不想为、不善为、不敢为的现象不同程度地存在，腐败问题还有发生。我们一定正视困难、直面挑战，坚持抓重点、补短板、强弱项、防风险，努力把各项工作做得更好。

二、 2018年重点任务

党的十九大指出，中国特色社会主义进入了新时代，这是我国发展新的历史方位。新时代的湖州，正处在重大战略机遇期、经济走势上扬期、高端要素加速集聚期，“两山”红利持续释放，交通优势加快形成，发展速度质量同步提升，干部群众精神状态更加振奋，各种有利因素向湖州汇聚。同时，发展中仍然面临着不少风险和挑战，城市间的竞争发生深刻变革，前进道路上有许多关口亟待攻克和突破。我们必须始终坚持高质量、竞争力、现代化的总体发展导向，把质量效益作为核心追求，把赶超争先作为根本任务，把改革创新作为源头活水，把特色优势作为最大本钱，把实干攻坚作为工作常态，把为民清廉作为政府铁律，奋力谱写新时代中国特色社会主义事业湖州新篇章。

今年是全面贯彻党的十九大精神的开局之年，是改革开放40周年，是决胜全面建成小康社会、实施“十三五”规划承上启下的关键一年。做好今年政府工作，总的是要高举习近平新时代中国特色社会主义思想伟大旗帜，全面贯彻党的十九大精神和中央以及省、市委各项决策部署，坚持稳中求进工作总基调，坚持新发展理念，紧扣社会主要矛盾变化，按照高质量发展的要求，坚定不移沿着“八八战略”指引的路子走下去，坚定不移践行习近平总书记“绿水青山就是金山银山”理念和“一定要把南太湖建设好”的重要指示精神，坚定不移一张蓝图绘到底，大力弘扬红船精神、浙江精神，持续深化“四新”主题实践，全力推进“六重”工作，打好防范化解重大风险、低收入百姓增收、污染防治三大攻坚战，统筹推进稳增长、促改革、调结构、惠民生、防风险各项工作，扎实推进全省“两个高水平”建设在湖州的生动实践，奋力当好践行“两山”理念的样板地、模范生，为加快赶超、实现“两高”奠定更加坚实的基础。

综合考虑各方面因素，建议今年全市经济社会发展的主要预期目标为：全市生产总值增长8%以上；固定资产投资增长11%；社会消费品零售总额增长10%；外贸出口保持稳定增长，继续提升占全省的份额；财政总收入和地方财政收入均增长8%以上；城镇新增就业6.5万人，登记失业率控制在3.5%以内；城镇、农村居民人均可支配收入分别增长8%以上和8.5%；研究与试验发展经费支出相当于地区生产总值的比例达到2.7%；节能减排降碳完成省下达任务。

重点做好以下工作：

（一）聚焦实体经济，加快构建现代产业体系

推动制造业向中高端迈进。深入推进“中国制造2025”试点示范城市建设，运行好“八个一”重点产业推进机制，加快绿色智能制造标准体系建设，争创“中国制造2025”国家级示范区。加快提升新型动力电池、高端绿色家居等优势产业，大力发展以数字经济为核心的新经济，确保规上工业增加值增长8%以上，确保战略性新兴产业增长快于规上工业。突出纺织、建材、蓄电池、金属加工、家具竹木、电梯电机等重点领域，落实创新升级、整合优化、集聚入园、有序退出等举措，淘汰100家以上企业的落后产能，建设小微企业园区15家，新建标准厂房150万平方米，全面加快传统制造业改造提升步伐。加快推动大数据、云计算、人工智能等与制造业融合发展，新增上云标杆企业20家、工业机器人1000台。大力开展品质革命，推进质量标准品牌建设，培育“品字标”企业30家，新增“浙江制造”标准30项，争创全国质量强市示范城市。

大力发展现代服务业。推进国家全域旅游示范区、国家级旅游业改革创新先行区建设，实施以“个十百千万”工程为主体的“龙腾计划”，推动太湖龙之梦项目顺利开业，确保旅游总收入增长10%。深化全国内河水运转型发展示范区建设，促进河海联运、水陆一体，力争内河集装箱吞吐量突破50万标箱。完善城乡物流体系，加快市农产品物流集散中心等项目建设。推进餐饮行业“厨房革命”，打造“放心消费在湖州”品牌。推动乡镇商贸振兴发展、社区商贸提升发展和线上线下融合发展，促进商贸业转型升级。大力培育和扶持创意设计、影视动漫、手游电竞等文化产业。坚持“房子是用来住的，不是用来炒的”定位，强化科学调控，促进房地产业平稳健康发展。

深化农业供给侧结构性改革。全面落实粮食安全责任制，稳定粮食产能，确保供给安全。实施现代渔业绿色发展和茶产业提升行动，整合特色水果、蔬菜、笋竹、花卉苗木等优势产业向园区集中，引领农业生态高效发展。大力发展林下经济。培育做大乡村旅游、精品民宿、农村电商等新业态。推进国家特色农产品优势区创建，建设一批省级现代农业园区、特色农业强镇，发展田园综合体和“星创天地”。大力培育新型农业经营主体和农业品牌，新增市级以上示范性家庭农场50家、市级农业龙头企业15家、新型职业农民1800名、省级以上农业品牌10个。完善农技推广服务体系，推进“互联网＋农业”，建成20个省级现代农业科技示范基地。

（二）聚焦创新创业，大力汇聚发展新动能

以最实的举措扩大有效投资。强化招商引资“一号工程”，完善考核评价办法，落实倒逼激励措施，推动各园区平台“70%的人员、 70%的精力”用于招商引资和项目推进，确保浙商回归省外到位资金增长10%以上，力争实到外资11亿美元。实施市县（区）长项目工程，主抓一批五十亿、二十亿大项目好项目。主攻工业项目特别是中高端制造业项目的引进推进，确保亿元以上工业项目新开工超过170个、竣工超过100个。深入实施和全面落实促进有效投资的各项政策措施，确保民间投资、重大产业项目投资、高新技术产业投资均增长12%以上，努力实现更高质量、更好效益、更可持续的投资增长。

用最大的力度集聚创新资源。以国家创新型城市建设为引领，实施“1+N”人才强市新政和新一轮南太湖人才工程，全面落实促进大学生就业创业等政策措施，瞄准国内外高校院所集中的区域，精准对接人才资源、创新要素，新引进大学生等各类人才6万名以上、“国千”和“省千”人才20名以上、领军型创新创业团队和人才项目200个左右，新增与大院名校合作共建的创新载体15家。促进湖州师院、湖州职院等地方高校办学与产业发展深度融合，深化与浙江大学等市外高校的合作。加快湖州科技城等重大创新平台建设，高水平打造产业创新服务综合体。鼓励民间资本利用库存楼宇、闲置厂房等存量资源发展众创空间，新增各类众创空间50家。大力推进创新成果转化应用，确保每万人发明专利拥有量达到26件。

靠最好的环境培育企业主体。大力弘扬新时代湖商精神和工匠精神，保护企业家财产权益、创新权益和自主经营权，营造更加优越的营商环境。落实各项降成本、促发展政策措施，有效运作政府产业引导基金，统筹用好涉企专项资金。加大企业梯度培育力度，力争“金象金牛”企业达到18家，启动新一轮“高技术高成长”企业培育计划，新增国家重点扶持高新技术企业90家，完成“个转企” 950家、“小升规” 400家、“下升上” 180家。实施“五年倍增行动”计划，推动企业股改上市和并购重组，力争完成股改100家，新增上市公司6家、挂牌企业20家。推动建筑企业晋升资质、做强做大，力争建筑业总产值增长20%以上。开展第四次全国经济普查。

（三）聚焦深化改革，持续释放制度红利

以深化“最多跑一次”改革促进政府自身改革。深化行政审批制度改革，继续大幅减少审批事项，大幅缩短审批服务时间，大幅下放审批权限，大幅提升网上审批能力，大幅降低审批服务费用。全面推广“一窗受理、集成服务”，确保所有民生事项和企业事项实现“一次办结”，其中80%以上开通网上办理、 50%以上的民生事项实现“一证通办”。全面推行企业投资项目承诺报备制改革，进一步减少前置环节，加强事中事后监管，力争项目受理到开工许可的审批时限全省最短。有效运行市大数据管理中心，打破“信息孤岛”，基本实现数据共享，提升“互联网＋政务服务”水平。深化公共资源交易集中监管体制改革，进一步规范招投标行为。积极稳妥推进经营类事业单位改革。

以绿色金融改革创新撬动投融资领域改革。坚持绿色金融支持绿色产业、服务实体经济导向，丰富产品、做大规模，确保绿色贷款增速快于全部贷款。推动设立保险公司、民营银行、财务公司等总部型法人金融机构，培育私募基金、股权投资等新业态，推进湖州银行改革发展，加快建设太湖绿色金融小镇、长三角金融后台服务基地、长兴太湖资本广场等集聚平台，确保新增社会融资规模1000亿元以上。打好防范化解重大风险攻坚战，严厉打击非法违规金融活动，继续加强银行不良资产处置和企业资金链担保链风险化解，确保信贷资产质量位居全省前三位。严控政府性债务总量，进一步降低成本、优化结构，确保政府债务率继续下降。深化国企国资改革，狠抓主业发展、效益提升和资产证券化，加大经营性资产整合盘活力度，推动国有企业做强做大、国有资产保值增值。

以“标准地”改革和“五未”土地处置助推要素配置市场化改革。推行企业投资项目“标准地”供地模式，政府设标出让、按标监管，企业对标竞价、照标履约，让有限的新增建设用地资源发挥出最佳效益。深化“亩均论英雄”改革，强化企业分类综合评价结果应用，对批而未供、供而未用、用而未尽、建而未投、投而未达标的建设用地进行专项处置，力争工业亩均税收、单位建设用地生产总值均提高10%，盘活存量建设用地6500亩。开展全域土地综合整治，推进矿地综合利用，科学利用低丘缓坡，严格落实耕地占补平衡。

（四）聚焦对外开放，努力拓展发展格局和空间

主动融入开放大战略。全面对接“一带一路”、长江经济带，开展外向型经济发展提升行动、外向型项目招引行动、跨境电商扩强行动。抢抓“上海2035”规划启动实施的机遇，谋划打造沪湖绿色智造大廊道，加快融入上海同城化都市圈。推动南浔等地更好发挥接轨上海的桥头堡作用。紧密对接宁杭生态经济带、大湾区大花园大通道大都市区、运河文化带等规划建设，进一步凸显湖州在区域发展中的战略地位。全力办好首届联合国世界地理信息大会以及国际锂电池峰会、世界木地板大会、国际乡村旅游大会，向世界展现美丽开放的湖州形象。深化与吉林省白山市等地的对口合作，做好对口支援、东西部扶贫协作以及外事侨务等工作。

加快建设开放大通道。全面实施交通“五年百项千亿”大会战，加速形成接轨沪杭、联通苏皖的大通道，构建外通、内畅、互联的现代综合交通网络，打造全国区域重点联运枢纽城市。实施高铁“三线”攻坚战，全速建设商合杭项目，全线开建沪苏湖项目，力争开工湖州至杭州西项目，加快推进安吉、南浔等配套场站建设，加速实现县区高铁全覆盖。完成宣杭铁路电气化改造，开展中心城市及杭德轨道交通项目前期。加快杭宁高速公路改扩建、杭州绕城高速公路西复线等工程建设，推进织新高速公路、苏台高速公路联络线等项目前期。开工建设申嘉湖高速公路安吉孝源至唐舍段、浙北高等级航道网集装箱运输通道等项目，加快推进京杭运河整治、长湖申航道西延等项目建设。

全力打造开放大平台。高起点谋划建设南太湖绿色发展新区，提升各类集聚区、开发区、高新区、度假区承载能力，加快形成10个万亩大平台的空间布局，争创国家级产业平台。在国家级开发区、高新区开展“证照分离”改革，实施重大平台项目准入新标准，深化“区域能评＋区块能耗标准”“区域环评＋环境标准”改革，构筑产业平台体制机制新优势。全面提升特色小镇产业、文化、旅游、社区等融合功能，打造全面践行新发展理念的高端平台。加快建设国际产业合作园，设立B型保税物流中心。（五）聚焦生态文明，不断提升城乡人居环境

深化生态文明建设领域改革创新。按照“样板地、模范生”的要求，深入推进生态文明先行示范区、“两山”实践创新基地建设，争创践行“两山”理念先行示范区。完善立法、标准、体制“三位一体”的生态文明制度体系，加强生态文明领域地方性法规建设，制订市级地方标准15项以上，完成环保机构监测监察执法垂直管理制度改革，推进自然资源资产产权制度改革，建立健全国有森林资源有偿使用制度，落实“河长制”“湖长制”“林长制”等制度。建立促进绿色生产生活的激励与约束机制，加快构建“生态＋电力”模式，全面推行中心城市单位生活垃圾强制分类，创建国家节水型城市，实现市区公交车电动化全覆盖。大力发展绿色建筑，稳步推进装配式建筑。

打好污染防治攻坚战。深入实施“811”美丽湖州建设行动，全域推进美丽湖州建设。开展10条重点河道区域综合治理，创建100个“污水零直排区”，打造1000条美丽生态河道，完成15万亩渔业养殖尾水治理，确保达到或优于Ⅲ类水质的县控及以上断面比例、水环境功能区达标率、县级以上城市集中式饮用水水源地水质达标率均保持100%，“五水共治”再夺大禹鼎。打好蓝天保卫战，开展“蓝天杯”考核，推进PM2.5和臭氧协同治理，实施机动车尾气排放遥感监测，全面禁止秸秆、垃圾露天焚烧，加快推进煤炭减量替代，推动禁止销售燃放烟花爆竹向更广区域拓展，深化挥发性有机物污染治理，确保市区PM2.5浓度下降到每立方米40微克以内、空气质量优良率达到72%以上。启动第二次全国污染源普查。全面开展土壤污染调查，逐步推进污染地块分类治理，完成南太湖垃圾焚烧发电厂四期、固体废物综合利用处置场项目建设。坚持总量压缩、自用为主，从紧从严、依法依规管理矿山，深化全国绿色矿业发展示范区建设，基本完成重点废弃矿山生态修复。

加强城市规划建设管理。完成“湖州城市总规2035”编制，开展第三次全国土地调查，启动新一轮土地利用总体规划编制，推进“多规合一”，建成市规划展览馆。坚持中心城区和长兴、南浔“一体两翼”组团发展，推动德清、安吉组团协同发展，提高全域城市化水平。统筹推进滨湖一体化开发建设，整体提升小梅山核心区块，基本建成湖长二通道，进一步扮靓南太湖65公里黄金岸线。抓好老城有机更新和新区开发建设，有序推进老旧居住区块征收改造，启动内环道路快速化改造，基本贯通湖山大道，建成五一大桥。深入推进“三改一拆”，完成城中村改造任务，争创基本无违建市。以生态修复、城市修补为抓手，改善城市生态环境，增强城市宜居性。巩固提升全国文明城市创建成果，全面实施中心城市精细化管理。推进物联网安防小区建设，规范和提升小区物业管理。加快智慧城市建设，实施市民卡工程，推进“雪亮工程”建设。深入实施小城镇环境综合整治。

打造实施乡村振兴战略示范区。实施产业提升、美丽建设、人文发展、乡村治理、民生优化、制度完善等“六大行动”，高水平创建农业全面现代化、环境全域美丽、生活全民幸福、要素全效流动的乡村振兴示范区。推动美丽乡村全方位提档升级，打造180个A级景区村庄，力争市级美丽乡村基本实现全覆盖。启动实施促进农民增收三年行动计划和新一轮促进低收入农户全面发展行动，努力增加农民收入。全面建成四大重点水利工程，加快推进三大后续工程，基本建成东苕溪绿道。加强农田水利基础设施建设，启动病险水库山塘“清零”行动。提升改造农村公路130公里，争创全国美丽公路示范市。统筹推进农村文化建设和乡村治理，打造100个自治法治德治相结合的善治示范村。全面完成农村“三权”确权登记颁证，稳步开展“活权”改革，深化农村集体资产股份合作制改革，推进承包地、宅基地“三权分置”，推动集体经营性建设用地入市改革，深化“三位一体”农合联建设。（六）聚焦民生福祉，切实增强人民群众获得感、安全感

提高居民收入和社会保障水平。精准帮扶困难群体就业，完善劳动关系协商机制，统一城乡失业保险政策，完善多层次社会救助机制，推动低保和扶贫政策的有效衔接，健全低保扩面提标机制，打好低收入百姓增收攻坚战。稳步提高退休人员养老保险待遇水平。完善城乡一体化基本医疗保险政策，探索长期护理保险制度。加快养老机构和养老床位建设，推动医养结合。加强妇女儿童权益保障，加大对重病和重残儿童救助力度，确保自闭症、先天性耳聋等患儿全部得到及时救治。推进住房制度改革，进一步扩大住房公积金覆盖面和受益面。大力发展红十字、残疾人、慈善等事业。

加快社会事业改革发展。大力弘扬社会主义核心价值观，选树“最美”典型，让“红马甲”“黄丝巾”等更多正能量温暖湖城。实施新一轮教育布局调整，推进普通高中质量提升工程，确保高考一段上线率有新提高。实施新一轮学前教育发展行动计划，推动义务教育优质均衡发展，完成中职教育现代学徒制改革试点。发展民办教育，推动与知名高校合作办学。推进健康湖州建设，争创健康中国示范区和综合医改先进市。全面推动医疗机构布局优化，启动市中医院迁建等重点项目建设。深化公立医院综合改革，高水平推进医联体建设，加快构建分级诊疗制度。提升智慧健康水平，强化医疗卫生服务能力建设，持续改善群众就医体验。推进人口计生服务管理转型。深入实施文化惠民工程，高标准打造“城市书房”“城市客厅”“城市阳台”等文化品牌，支持原创精品创作。推进文物活化利用，加强溇港圩田、桑基鱼塘等文化遗产的保护、利用和展示，讲好“丝、茶、笔”等文化故事，办好第九届湖笔文化系列活动。高质量办好第十六届省运会，广泛开展“全民健身与省运同行”群众性体育活动，推动竞技体育、群众体育和体育产业协调发展。

打造共建共治共享的社会治理格局。落实“谁执法谁普法”“谁服务谁普法”的普法责任制，增强全社会法治信仰。畅通群众诉求反映渠道，完善人民调解、行政调解、司法调解无缝对接机制，加快仲裁事业发展，注重矛盾纠纷的源头防范和多元化解。加强“基层治理四平台”能力和全科网格规范化建设，夯实社会治理基层基础。以幸福邻里中心建设为切入点，推动社区、社会组织、社会工作者有序参与社会治理，促进政府治理和社会调节、居民自治良性互动。健全守信联合激励和失信联合惩戒机制，营造“诚实守信光荣、背约失信可耻”的社会信用环境。坚持“防为主、防为上”，开展扫黑除恶专项斗争，打造社会治安防控体系升级版，提高社会治理社会化、法治化、智能化和专业化水平。强化从田头到餐桌的全过程监管和智慧监管，保障“舌尖上的安全”，争创省级食品安全市和国家农产品质量安全市。深化安全生产领域改革发展，严格落实安全生产责任制，创新监管机制和手段，清单化排查和整治事故隐患。推进气象现代化，加强应急管理，增强防灾减灾能力。加快构建综合性、全方位、系统化的城市安全发展体系，全力夺取平安金鼎。落实军民融合发展战略，推进人民防空建设，实现省级双拥模范城“八连冠”。扎实做好民族、宗教等工作。

各位代表！增进民生福祉是发展的根本目的。经过广泛征集、深入论证，市人民政府向本次大会提交今年民生实事十二方面的候选项目，提请票决选出其中十个方面。民生实事项目确定后，我们将以高度负责的态度、扎实有力的举措，认真抓好推进和实施，确保群众真正得到实惠。

三、全面加强政府自身建设

越是重任在肩，越要自身过硬。我们要不断深化“四新”主题实践，坚持团结、奋发、担当、高效、干净，全面加强政府自身建设，着力提升政府执行力和公信力，努力建设人民满意的服务型政府。

（一）始终坚持不忘初心、牢记使命，着力打造为民政府。切实增强“四个意识”，坚定“四个自信”，坚决同以习近平同志为核心的党中央保持高度一致，不折不扣贯彻落实好省委、省政府和市委各项决策部署。扎实开展“不忘初心、牢记使命”主题教育，坚定理想信念，坚守人民立场，永葆蓬勃朝气，勇立时代潮头，带着初心和使命干好政府工作。把人民对美好生活的向往作为奋斗目标，牢记群众之事无小事、企业之事无小事，大兴调查研究之风，走进基层、走进一线、走进矛盾，从一件件“关键小事”抓起，切实解决发展所需、群众所盼的实际问题，以改革发展的实际成效取信于民、造福于民。

（二）始终坚持大抓落实、大干实事，着力打造有为政府。优化政府机构设置和职能配置，深化机构改革，加快形成职责明确、依法行政的政府治理体系。以奋发有为的精神状态、勤勉务实的工作作风，出实招、办实事、求实效。坚决不搞虚假政绩、不搞数字游戏，始终突出“六重”工作，继续排出一批于当前迫切、对长远有利的重大事项，一件一件抓好落实。进一步完善抓落实的工作机制，确保事事有人干、人人有责任。加强督促检查和绩效管理，全面整治庸政懒政怠政，加大对工作落实不力、效能低下、敷衍塞责、弄虚作假等行为的追责问责力度。建立健全正向激励和容错纠错机制，为敢闯敢试者撑腰，给改革创新者鼓劲，让广大公务人员愿干事、敢干事、能干事、干成事。

（三）始终坚持依法行政、公开透明，着力打造法治政府。自觉接受人大依法监督和政协民主监督，主动接受司法监督、社会监督和舆论监督。严格遵守宪法法律，深化法治政府建设，推动重点领域立法，加强行政执法规范化建设，完成行政复议体制改革，更好发挥政府法律顾问制度的作用。认真听取民主党派、工商联、无党派人士和人民团体的意见，加强新型智库建设，持续深化“开门听建议”。严格执行行政决策法定程序，完善市人大代表、市政协委员列席市政府常务会议等制度，切实做到“开门作决策”。坚持以公开为常态、不公开为例外，全面推进政务公开，将行政行为放在阳光之下，全面落实“开门晒结果”。

（四）始终坚持防微杜渐、干净干事，着力打造清廉政府。坚定不移推动全面从严治党，严明纪律规矩，坚持“一岗双责”、以上率下，层层压实责任，狠抓清廉湖州建设各项任务落实，持续提升政府系统从严管党治党水平。严格执行贯彻落实中央八项规定实施细则的办法，坚决防止隐形变异的“四风”问题反弹回潮。牢记廉洁之事无小事，全力支持纪检监察机关履行职责，严查侵害群众利益的不正之风和腐败问题，切实做到干部清正、政府清廉。完成事业单位和国有企业公务用车制度改革，进一步规范机关事业单位资产管理。推进审计体制改革，在政策落实、权力运行、公共资金、重大工程、自然资源资产等领域，全面加强审计监督，不断完善权力运行的制约监督机制。

各位代表！新时代开启新征程，新使命呼唤新担当。让我们高举习近平新时代中国特色社会主义思想伟大旗帜，在中共湖州市委的领导下，团结一心、锐意进取、脚踏实地、埋头苦干，为加快赶超发展，高质量建设现代化生态型滨湖大城市、高水平全面建成小康社会而努力奋斗！
